# Discovery and characterization of small molecule Rac1 inhibitors

**DOI:** 10.18632/oncotarget.16656

**Published:** 2017-03-29

**Authors:** Jamie L. Arnst, Ashley L. Hein, Margaret A. Taylor, Nick Y. Palermo, Jacob I. Contreras, Yogesh A. Sonawane, Andrew O. Wahl, Michel M. Ouellette, Amarnath Natarajan, Ying Yan

**Affiliations:** ^1^ Department of Radiation Oncology, University of Nebraska Medical Center Omaha, Nebraska, United States of America; ^2^ Eppley Institute for Research in Cancer and Allied Diseases, University of Nebraska Medical Center Omaha, Nebraska, United States of America; ^3^ Holland Computing Center University of Nebraska-Lincoln Omaha, Nebraska, United States of America; ^4^ Department of Internal Medicine, University of Nebraska Medical Center Omaha, Nebraska, United States of America; ^5^ Department of Biochemistry and Molecular Biology, University of Nebraska Medical Center Omaha, Nebraska, United States of America; ^6^ Department of Pharmaceutical Sciences, University of Nebraska Medical Center Omaha, Nebraska, United States of America; ^7^ Department of Genetics, Cell Biology and Anatomy, University of Nebraska Medical Center Omaha, Nebraska, United States of America

**Keywords:** GTPase, Ras-related C3 botulinum toxin substrate 1 (Rac1), Cdc42, Ras homolog gene family member A (RhoA), inhibitor

## Abstract

Aberrant activation of Rho GTPase Rac1 has been observed in various tumor types, including pancreatic cancer. Rac1 activates multiple signaling pathways that lead to uncontrolled proliferation, invasion and metastasis. Thus, inhibition of Rac1 activity is a viable therapeutic strategy for proliferative disorders such as cancer. Here we identified small molecule inhibitors that target the nucleotide-binding site of Rac1 through *in silico* screening. Follow up *in vitro* studies demonstrated that two compounds blocked active Rac1 from binding to its effector PAK1. Fluorescence polarization studies indicate that these compounds target the nucleotide-binding site of Rac1. In cells, both compounds blocked Rac1 binding to its effector PAK1 following EGF-induced Rac1 activation in a dose-dependent manner, while showing no inhibition of the closely related Cdc42 and RhoA activity. Furthermore, functional studies indicate that both compounds reduced cell proliferation and migration in a dose-dependent manner in multiple pancreatic cancer cell lines. Additionally, the two compounds suppressed the clonogenic survival of pancreatic cancer cells, while they had no effect on the survival of normal pancreatic ductal cells. These compounds do not share the core structure of the known Rac1 inhibitors and could serve as additional lead compounds to target pancreatic cancers with high Rac1 activity.

## INTRODUCTION

The Rho family of guanosine triphosphatases (GTPases) are important regulators of diverse cellular functions including cytoskeleton organization, cell cycle progression and motility [1, 2]. Rho GTPases can exist either in an active GTP-bound state or in an inactive GDP-bound state. The transition between these two states is regulated by guanine nucleotide exchange factors (GEFs) and GTPase-activating proteins (GAPs). GEFs promote Rho GTPase activation by facilitating the exchange of GDP for GTP. Once activated, Rho GTPases can interact with their downstream effectors to activate various downstream signaling pathways. GAPs terminate the activity of Rho GTPases and downstream signaling by promoting GTP hydrolysis and returning Rho GTPases to an inactive GDP-bound state [3, 4]. Rac1, Cdc42, and RhoA are Rho GTPase family members and have many overlapping cellular functions including cytoskeleton reorganization, cell cycle regulation, motility and cell survival. Accumulating evidence has implicated these Rho GTPases as regulators of many aspects of tumorigenesis including proliferation, invasion and migration of cells [5–7].

Ras-related C3 botulinum toxin substrate 1 (Rac1), a member of the Rho family GTPases, plays a critical role in cell migration, cell proliferation, cell survival and malignant transformation [8–10]. Rac1 is activated by a variety of stimuli, including receptor tyrosine kinases, G-protein-coupled receptors, and integrins, by conveying signals through Rac-GEFs. Deregulation of upstream activators due to activating mutations or increases in the levels of growth factors (EGF, HGF, PDGF) leads to increased Rac1 activity [11]. Consequently, Rac1 activity has been implicated in a number of cancers, including breast, colon, prostate, and pancreatic cancer [12–15]. In more than 70% of pancreatic cancer, overexpression of Rac1 has been observed. Additionally, its hyper-activation, caused by overexpression of two of its GEFs, Tiam1 and Vav1, has been previously documented [15–17]. Overexpression of Vav1 has also been associated with poor prognosis of pancreatic cancer patients [17, 18]. In a mouse model of pancreatic cancer, Rac1 knockdown was shown to reduce tumor formation and prolong survival [19]. Together, these studies suggest that targeting Rac1 is a viable therapeutic strategy for a number of cancers.

Rac1 contains a G (guanine nucleotide-binding)-domain, which consists of a six-strand β-sheet surrounded by α-helices and a 13-residue insertion unique to the Rho GTPase family. This encompasses its four functional regions, Switch I, Switch II, Insert region, and Hypervariable region. The Switch regions are responsible for many of Rac1's molecular interactions with Switch I primarily interacting with downstream effectors while Switch II interacts with GEFs to regulate Rac1 activation [20]. Although there is high sequence and structural homology between Rac1, Cdc42, and RhoA, interactions with effectors and GEFs are selective among these GTPases. Several inhibitors have been developed to target the GEF-binding site of Rac1 [21–26]. Many GEFs such as Vav1 and Tiam1 that are overexpressed in pancreatic cancer are known to promote Rac1 activation [15–17, 27]. Although different GEFs bind to Rac1 and activate different signaling cascades, the majority of Rac1-specific GEFs share a common binding site [28–30]. The nucleotide-binding site in Rac1 is considered undruggable and to date two inhibitors, MLS000532223 and EHT1864, have been reported to inhibit Rac1 activity by altering nucleotide binding to Rac1 [31, 32]. However, their exact mechanism of action is yet to be fully defined. Nevertheless, these studies indicate that small molecules can be used to disrupt nucleotide binding to Rac1. To this end, we performed a structure-based *in silico* high-throughput screening to identify small molecule inhibitors that target the nucleotide-binding site on Rac1. Here we report the identification of two potential small molecules with core structures that are dissimilar to previously reported Rac1 inhibitors that perturb nucleotide-binding to Rac1. The two inhibitors, #1 and #6, are selective for Rac1 and reduce cell growth and migration in pancreatic cancer cell lines.

## RESULTS

### Identification and validation of Rac1 GTPase inhibitors

To identify novel Rac1 inhibitors that target the nucleotide-binding site, a virtual high-throughput screen was performed using the 100,000-member ChemBridge chemical library. Molegro Virtual Docker was used to dock compounds from the library against the crystal structure of Rac1 (PDB code: 3TH5). A docking sphere, radius 9Å, centered over the nucleotide-binding site was generated and the screen was executed using GPU accelerated algorithm under default settings. Compounds were ranked based on their re-ranked score and the top 1% of hits were selected for post-docking analysis. Post-docking analysis included the use of ACD Percepta software to assess ADMET and physicochemical properties of the hits. Following the post-docking analyses a set of 10 compounds were identified for experimental characterization.

**Figure 1:**
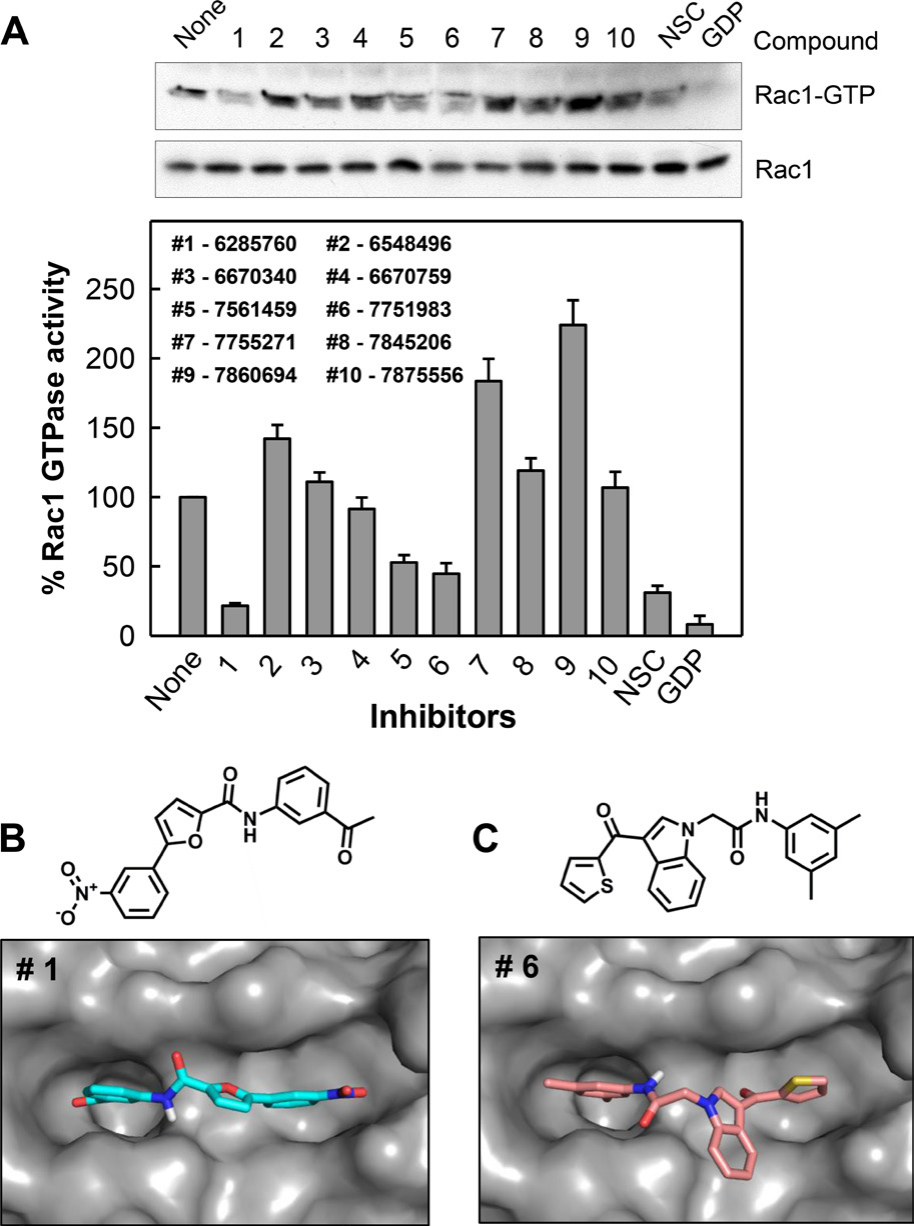
Identification of compounds #1 and #6 as inhibitors of Rac1 (**A**) The inhibitory effect on Rac1 activity by a panel of compounds identified in a virtual screen. CD18/HPAF cells were incubated with 10 μM of indicated compound for 2 h and Rac1 activity (Rac1-GTP) was determined using Rac1 GTPase assay. As positive controls, cells incubated with 100 μM NSC23766 *in vivo* for 2 h and lysate of log-phase growing cells incubated with 1 mM GDP for 15 min *in vitro* were included in the analysis. Upper panel: Rac1 activity (Rac1-GTP) in the samples were analyzed by Western blotting. Lower panel: Immunoblot densities of Rac1-GTP and Rac1 were quantified using ImageJ software and relative Rac1 activity versus total Rac1 was determined. Predicted binding modes for compounds #1 (**B**) and #6 (**C**) to the GTP-binding site of Rac1.

The set of 10 hit compounds were subjected to a cell-based assay to examine their ability to inhibit Rac1 activity in a pull-down assay previously reported by us [33, 34]. CD18/HPAF pancreatic cells were treated for 2 h with vehicle, 10 μM compound, or positive controls (100 μM NSC23766 or 1 mM of GDP) which have previously been shown to inhibit Rac1 activation by preventing GEF binding [21]. Active Rac1 (Rac1-GTP) was then pulled down using GST-tagged Rho GTPase binding domain (RBD) of PAK1 (p21-activated serine/threonine kinase) [35], and analyzed by Western blot analysis using a Rac1 specific antibody [33, 34]. Levels of Rac1-GTP (Rac1 activity) detected were then normalized to total Rac1 levels and represented as a bar graph in Figure 1A. This study shows that compounds #1, #5 and #6 inhibited Rac1 activity at levels comparable to NSC23766. It is important to note that the hit compounds were tested at 10-fold lower concentration as compared to the positive control NSC23766. From this, the two most potent, compounds #1 and #6, were selected for further studies.

The binding modes of compounds #1 and #6 were explored by additional docking experiments using Autodock Vina wherein the docking sphere was expanded to include all of Rac1. We observed that the majority of docked conformations for both compounds clustered within the nucleotide-binding pocket of Rac1. Figure 1B and 1C summarizes the most favorable docking conformation with the lowest energy of compound #1 (−8.0 kcal/mol) and #6 (-7.6 kcal/mol) and their chemical structures. Both compounds are positioned within the guanine recognition site of Rac1; however, neither is close enough to make significant contacts with the Switch II region of Rac1, which is involved with γ-phosphate binding [20]. The clustering of docked structures of both compounds to the nucleotide-binding site of Rac1 indicates that these compounds may act by disrupting nucleotide binding.

### Compounds #1 and #6 inhibit Rac1 complex formation with PAK1

**Figure 2:**
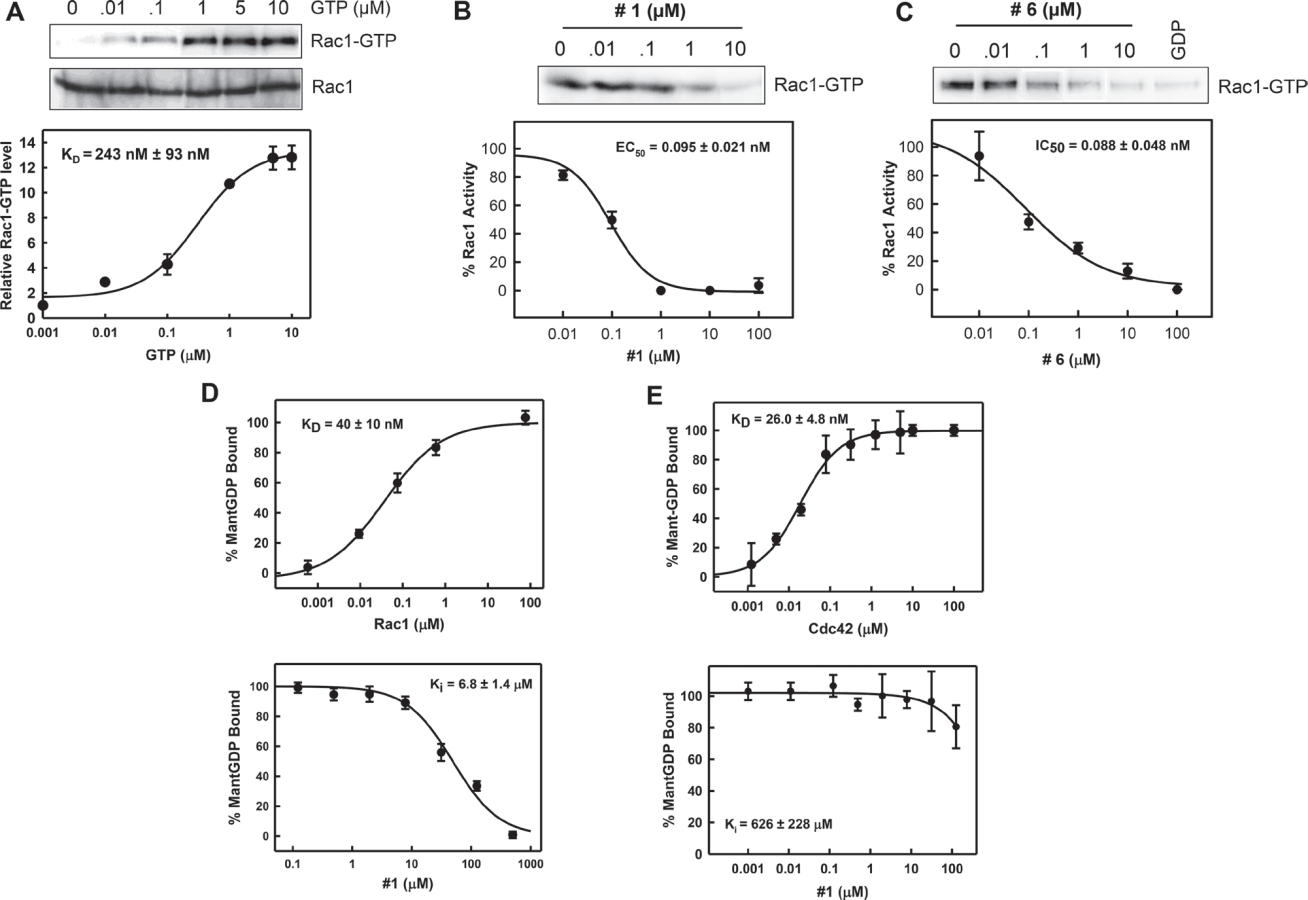
Compounds #1 and #6 block Rac1-PAK1 complex formation (**A**) Increasing concentrations of GTP-γS (GTP) were incubated with full-length Rac1 *in vitro* followed by pull-down of Rac1-GTP using GST-PAK1. Upper panel: Rac1-GTP and total Rac1 were visualized by immunoblotting. Lower panel: Data were fitted using a nonlinear least squares fit to a single site-binding model to determine apparent binding affinities. Rac1 (1 μM) was incubated with compound #1 (**B**) or #6 (**C**) at increasing concentrations for 1 h followed by a 10-min incubation with GTP. Rac1-GTP was then pulled-down using GST-PAK1. Active Rac1 levels were normalized to total Rac1 levels. Upper panel: Rac1-GTP was visualized by immunoblotting. Lower panel: Data were fitted using a nonlinear least squares fit to a single site-binding model to determine IC_50_ values. Results are shown as mean ± SD of two experiments. (**D**) Upper graph: Mant-GDP (100 nM) was incubated with increasing concentrations of recombinant Rac1 for 10 min. Binding of mant-GDP to Rac1 was monitored using fluorescence polarization (λ_ex_ = 360 nm, λ_em_ = 440 nm) assay. Lower graph: Increasing concentrations of compounds #1 were incubated with 1 μM Rac1 for 1 h for improved signal-to-noise. Mant-GDP was then added and fluorescence polarization was read after 10 min incubation. Values were normalized as percent bound. Data were fitted using a nonlinear least squares fit to a single site-binding model to determine K_i_. Results are shown as mean±S.D. of two sets of experiments done in triplicate. (**E**) Upper graph: Mant-GDP (100 nM) was incubated with increasing concentrations of recombinant Cdc42 for 10 min. Binding of mant-GDP to Cdc42 was monitored using fluorescence polarization (λ_ex_ = 360 nm, λ_em_ = 440 nm) assay. Lower graph: Increasing concentrations of compound #1 were incubated with 1 μM Cdc42 for 1 h for improved signal-to-noise. Mant-GDP was then added and fluorescence polarization was read after 10 min incubation. Values were normalized as percent bound. Data were fitted using a nonlinear least squares fit to a single site-binding model to determine K_i_. Results are shown as mean ± S.D. of two sets of experiments done in triplicate.

To further evaluate these compounds, we examined their effects on the *in vitro* formation of Rac1-PAK1 complex using purified recombinant proteins. For this analysis, we used full-length Rac1 and titrated increasing concentrations of GTP-γS (0.01 – 10 μM), a non-hydrolysable GTP analog. Active Rac1 (Rac1-GTP-γS) was then pulled-down using GST-PAK1 (RBD) (Figure 2A, upper panel). Active Rac1 was normalized to total Rac1 and showed a dose-dependent increase in binding to PAK1. The data was curve fitted and the apparent binding affinity was calculated to be 243 ± 93 nM (Figure 2A, lower panel). This is comparable to the K_D_ from a previous report using a similar assay [36], indicating that this assay will be suitable for studying formation of Rac1-PAK1 complex *in vitro*.

We next examined the effects of compound #1 and #6 on the formation of Rac1-PAK1 complex. Increasing concentrations (0.001 – 100 μM) of either compound #1 or #6 were incubated with Rac1 for 1 h and followed by additional incubation with GTP-γS and PAK1. As shown in Figure 2B, 2C, presence of either compound #1 or #6 resulted in a dose-dependent decrease in Rac1-PAK1 interaction with nearly a complete loss in interaction at 10 μM. Additionally, IC_50_ values, determined through curve fitting, were calculated for compound #1 and #6 as 95 ± 21 nM and 88 ± 48 nM, respectively (Figure 2B, 2C, lower panels). This data suggests that both compounds disrupt the formation of Rac1-PAK1 complex with low nanomolar potency.

### Compound #1 disrupts the interaction of Rac1 with mant-GDP

Our preliminary cell-based and cell-free studies suggested that both compound #1 and #6 could disrupt Rac1 binding to PAK1 and computational studies indicate this could be by disruption of nucleotide binding. We used a previously reported fluorescence polarization assay to study the effects of compounds #1 and #6 on nucleotide-binding. Fluorescently labeled GDP (mant-GDP) was incubated with increasing concentrations of recombinant Rac1 protein and binding of the nucleotide to Rac1 was analyzed using a fluorescence polarization assay by monitoring fluorescence polarization of mant-GDP (λ_ex_ = 360nm, λ_em_ = 440 nm). Results in Figure 2D (upper graph) showed a dose-dependent increase in fluorescence polarization of mant-GDP following addition of Rac1, indicating a dose-dependent binding of mant-GDP to Rac1. Using a nonlinear least squares fit to a single site-binding model (SigmaPlot 11.0) we calculated the apparent binding affinity of 40 ± 10 nM, which is comparable to previously reported K_D_ values for Rac1-nucleotide binding assays [37, 38]. To investigate nucleotide binding in the presence of compound #1, increasing concentrations (0.1–1000 μM) of compound #1 was incubated with Rac1 (250 nM) for 1 h followed by the addition of mant-GDP (100 nM), and fluorescence polarization was read after a 10-min incubation. With compound #1, we observed a dose-dependent decrease in fluorescence polarization, indicating loss of mantGDP binding to Rac1. These values were normalized as percent bound (Figure 2D, lower graph) and data was fitted using a nonlinear least squares fit to a single site-binding model, which yielded a K_i_ of 6.8 ± 1.4 μM. The loss of mant-GDP binding to Rac1 in the presence of compound #1 indicates that it acts by inhibiting nucleotide binding to Rac1. Due to fluorescence interference by compound #6, its effect on nucleotide binding could not be studied using this assay.

To validate the specific effect of compound #1 on the binding of mant-GDP to Rac1, we tested its effect on the binding of mant-GDP to Cdc42, the closest family member of Rac1. We first determined the affinity of Cdc42 with mant-GDP as described above. Results in Figure 2E (upper graph) showed a dose-dependent increase in binding of mant-GDP to Cdc42 with a binding affinity of 26.0 ± 4.8 nM. However, compound #1 was unable to inhibit the binding of mant-GDP to Cdc42 in a competition FP assay (Figure 2E, lower graph). Our attempts to establish a FP binding assay with mant-GDP and RhoA was not fruitful.

### Compounds #1 and #6 interferes with Rac1 activation following EGF stimulation

**Figure 3:**
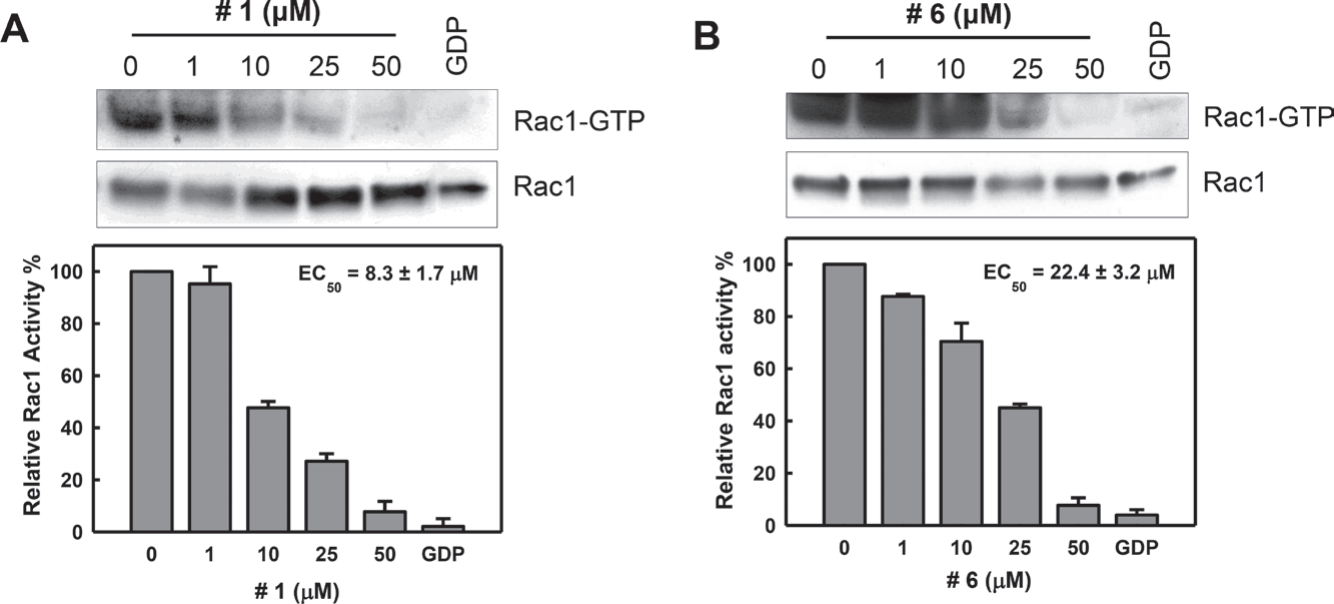
Inhibition of Rac1 activity in cells by compounds #1 and #6 CD18/HPAF cells were serum starved in the medium containing 0.3% FBS for 24 h, incubated for 2 h in the presence of compound #1 (**A**) or #6 (**B**) at the indicated concentrations and then stimulated with EGF (100 ng/ml) for 5 min. Upper panels: level of Rac1-GTP and total Rac1 in the samples were determined by Rac1 activity assay and immunoblotting, respectively. Lower panels: immunoblot densities of Rac1-GTP and Rac1 were quantified using ImageJ software and relative Rac1 activity versus total Rac1 was determined. The obtained data were fitted using a nonlinear least squares fit to a single site-binding model to determine the EC_50_. Results are shown as mean ± S.D. of two sets of experiments.

Since EGF (epidermal growth factor) is a well-known Rac1 activator in multiple cellular systems [17, 39, 40], we evaluated the effects of compounds #1 and #6 on Rac1 activation following EGF stimulation using a PAK1 pull-down assay. CD18/HPAF cells were treated with increasing concentrations (1–50 μM) of either compound #1 or #6 for 2 h followed by EGF stimulation and Rac1 activity was measured. Following treatment, both compound #1 (Figure 3A) and #6 (Figure 3B) diminished the EGF-induced Rac1 activation in a dose-dependent manner, with a nearly complete inhibition observed at 50 μM for both compounds. Additionally, EC_50_ values determined through curve fitting were 8.3 ± 1.7 μM for compound #1 and 22.4 ± 3.2 μM for compound #6 (Figure 3A, 3B, lower panels). In our hands, EHT-1864, which was previously shown to interfere with Rac1 nucleotide exchange [32], did not inhibit Rac1 activation in CD18/HPAF pancreatic cancer cells following EGF stimulation, as shown in Supplementary Figure 1A.

### Specificity for Rac1 by compounds #1 and #6 was determined in pancreatic cancer cells

Rac1, RhoA, and Cdc42, are the most extensively studied of the Rho GTPase family. They share many overlapping functions in cytoskeleton dynamics, motility, cell cycle progression, transcriptional regulation and cell survival [7]. All three GTPases are structurally similar, sharing a common G-domain fold and a 13-residue insertion characteristic of Rho GTPase family members. Cdc42 and RhoA also share high sequence homology, 70% and 57%, respectively, to Rac1 [41]. Since Rac1 is closely related to Cdc42 and RhoA GTPases, we compared the cellular effect of compounds #1 and #6 on the activities of Rac1, Cdc42 and RhoA.

**Figure 4:**
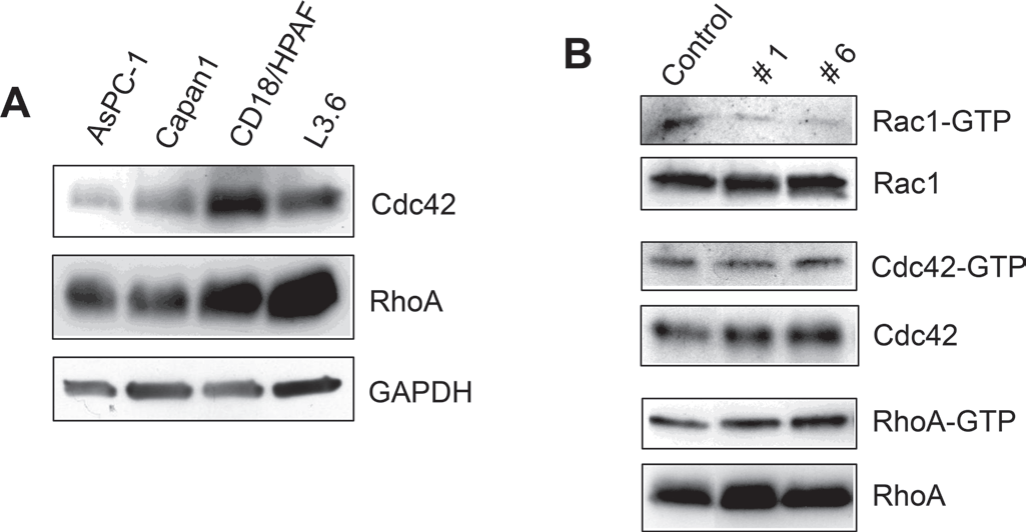
Effect of compounds #1 and #6 on the activities of Cdc42 and RhoA GTPases (**A**) Expression of Rho GTPases Cdc42 and RhoA in a panel of pancreatic cancer cell lines (**B**) CD18/HPAF cells were treated with 50 micromolar compound (#1 or #6) or vehicle control for 2 h. Cell lysates were then subjected to pull-down assays using agarose-immobilized GST-WASP, GST-Rhotekin, and GST-PAK1, to assess changes in GTP-bound levels of Rho GTPases Cdc42, RhoA, and Rac1, respectively. GTP-bound Cdc42, RhoA, and Rac1 were analyzed by Western blot analysis using specific antibodies. Total levels of Cdc42, RhoA, and Rac1 were also analyzed by Western blot and are included as controls.

To choose the best cell line for the study, we first assessed the expression of Cdc42, RhoA and Rac1 in a panel of pancreatic cancer cell lines [34]. CD18/HPAF cells showed high expression of all three GTPases (Figure 4A) and hence was selected for selectivity studies [34]. To compare the effect of compounds #1 and #6 on Rac1, Cdc42, and RhoA, CD18/HPAF cells were incubated with 50 μM of either compound #1, #6 or vehicle control (DMSO) for 2 h. GTP-bound Rac1, Cdc42, and RhoA were pulled-down using GST-PAK1, GST-WASP or GST-Rhotekin, respectively [21]. Results in Figure 4B showed that both compound #1 and #6 significantly inhibited Rac1 activity in CD18/HPAF cells compared to control, whereas they had no inhibitory effect on either Cdc42 or RhoA in CD18/HPAF cells. Additionally, neither compound affected the steady-state protein levels of the three Rho GTPases (Figure 4B). These results suggest that compounds #1 and #6 are selective for Rac1 GTPase in CD18/HPAF cells.

### Compounds #1 and #6 impede proliferation of pancreatic cancer cells

**Figure 5:**
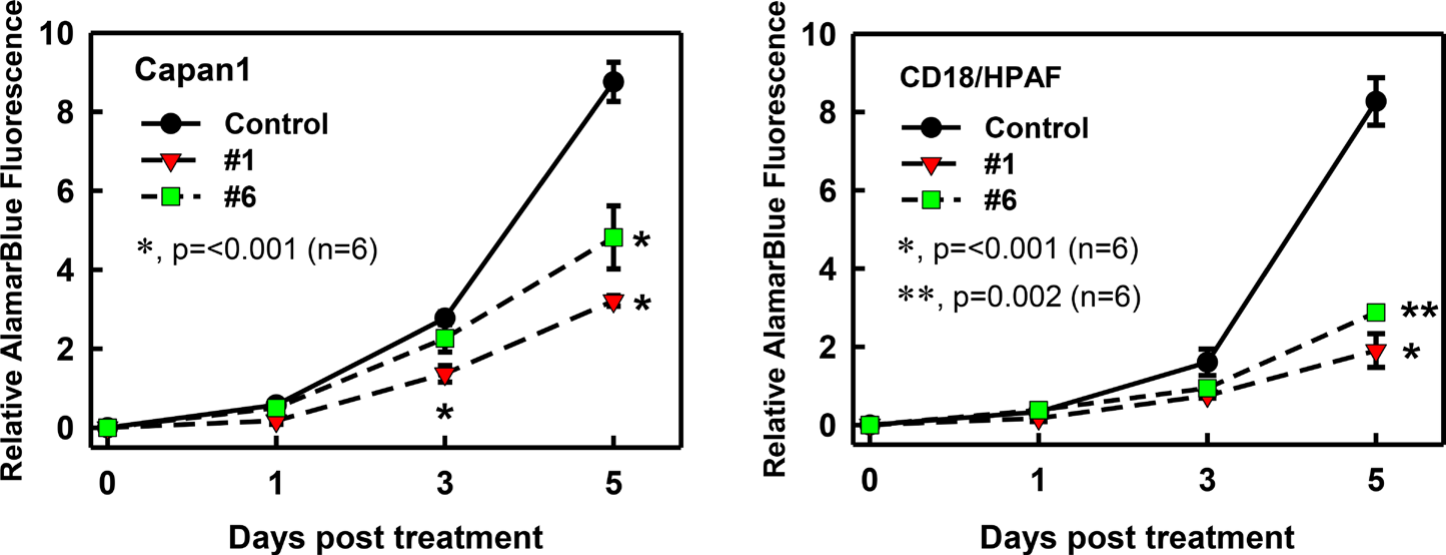
Effect of compounds #1 and #6 on growth kinetics of pancreatic cancer cells Capan1 and CD18/HPAF pancreatic cancer cells were used to test the effect of the compounds on growth kinetics. Cells (1.5 × 10^3^) were plated in 96-well plates, incubated with compound (25 μM) or vehicle DMSO for 0, 1, 3, and 5 days and then examined for the amounts of cells using AlamarBlue Assay described in the Materials and Methods. Results shown as mean ± S.D. of two sets of experiments done in triplicate. *p = < 0.001 (*n* = 6); **p = 0.002; significant difference between cells treated with compound (#1 or #6) and vehicle control.

Rac1 activity has been shown to be important for proliferation and cell cycle regulation of cancer cells [42, 43]. Hence we assessed the effect of the compounds on growth kinetics of pancreatic cancer cells using an AlamarBlue assay [44]. As shown in Figure 5, control-treated Capan1 and CD18/HPAF cells displayed a time-dependent increase in fluorescence intensity, detected using AlamarBlue Assay, indicative of exponential growth of these cells. However, incubation with compound #1 or #6 significantly inhibited the proliferation of these cells (*p = < 0.001, *n* = 6; **p = 0.002). In both cell lines, compound #1 showed a greater effect on cell growth when compared to compound #6. In Capan1 cells (Figure 5), a small but significant decrease in amounts of cells at day 3 was also observed with compound #1 (red triangles) compared to control cells (*p = < 0.001, *n* = 6). However, both compounds showed only a marginal decrease in CD18/HPAF cell proliferation at day 3 (Figure 5).

We also compared compound #1 with EHT-1864, an inhibitor previously shown to disrupt nucleotide binding to Rac family GTPases [32], for their effects on the proliferation of pancreatic cancer cells. We observed that compound #1 had a better cell growth inhibition profile when compared to EHT-1864 in pancreatic cancer cell lines. Briefly, AsPC-1 and CD18/HPAF cells treated with 25 μM compound #1 showed a ˜50% and ˜20% decrease in proliferation, respectively (Supplementary Figure 1B). However, 25 μM of EHT-1864 treatment failed to inhibit growth of CD18/HPAF cells and inhibited growth of AsPC-1 cells by ˜20%.

### Compounds #1 and #6 reduced migration of pancreatic cancer cells

**Figure 6:**
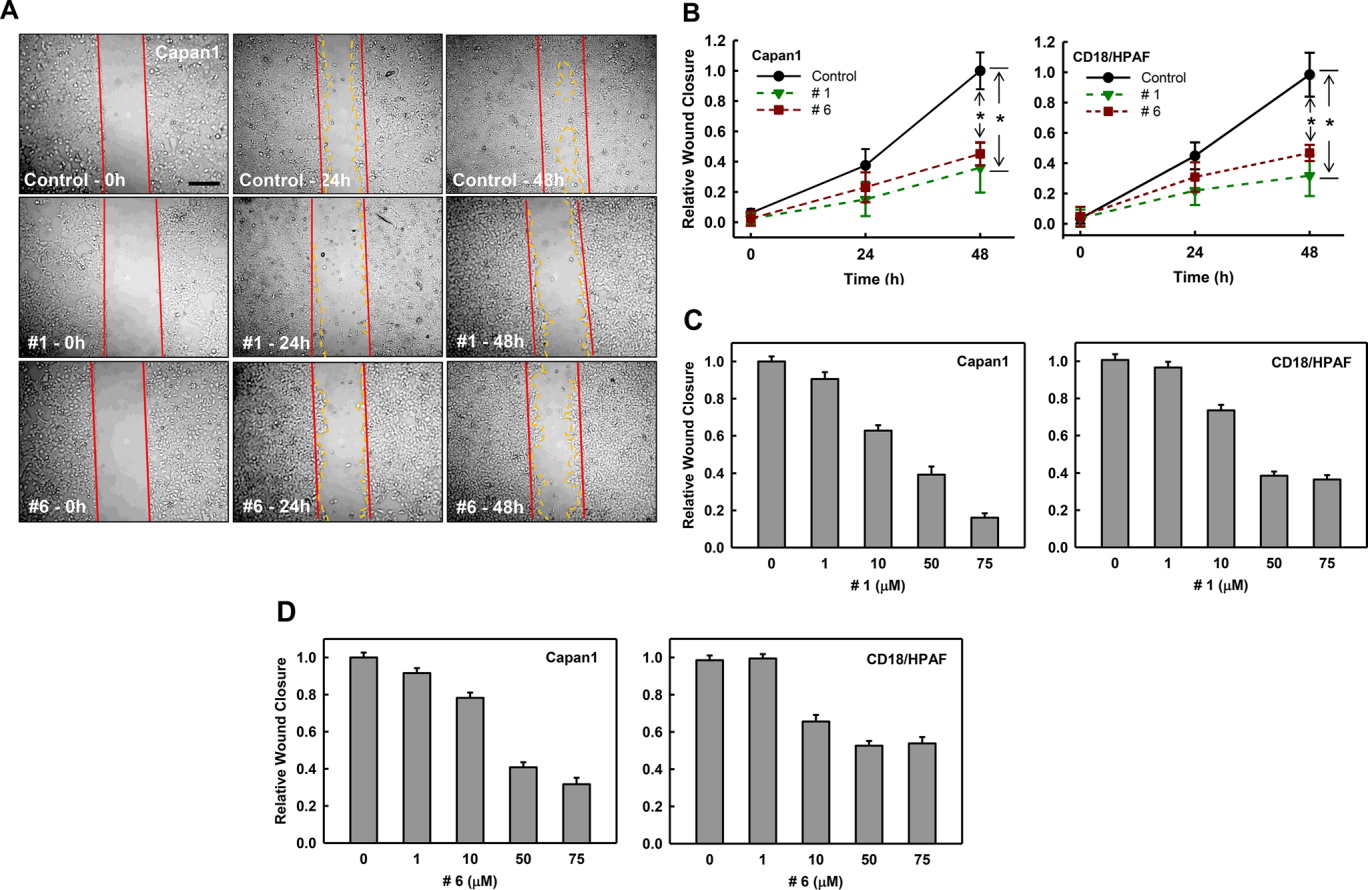
Effect of compounds #1 and #6 on mobility of pancreatic cancer cells Wounds were introduced to Capan1 and CD18/HPAF cells at 90% confluence using a pipette tip as described in Materials and Methods. The cells were incubated in the presence of DMSO control (0.1%), compound #1 (50 μM) or compound #6 (50 μM) and assessed for wound healing. (**A**) Representative images of cell migration of Capan1 following the indicated treatments. The images were taken at 0 h, 24 h, and 48 h post-wound. (**B**) Quantitation of cell migration of Capan1 (left panel) and CD18/HPAF (right panel) over time after compound or control treatment. Wound closure was quantified at 0 h, 24 h, and 48 h post-wound by measuring the remaining un-migrated area. Migration was normalized to relative wound closure in vehicle control and shown as mean ± S.D. from two sets of experiments in duplicate samples. *p = < 0.001 (*n* = 4), significant difference in cells exposed to compound #1 or #6 compared to vehicle control. (**C**) and (**D**) Cell samples with wounds were treated with increasing concentrations of compound #1 and #6 for 48 h and examined for their effects on mobility using wound-healing motility assay [65]. Quantitation of migration of Capan1 and CD18/HPAF cells following treatment with compound #1 (C). Quantitation of cell migration in Capan1 and CD18/HPAF cells following treatment with increasing concentrations of compound #6 (D). Wound closure was quantified by measuring the remaining un-migrated area. Migration was normalized to wound closure in the vehicle control and is shown as mean ± S.D. from two sets of experiments in duplicate samples.

Enhanced cell migration in tumor cells is a prerequisite for tumor invasion and metastasis [45]. Rac1 has been shown to regulate multiple downstream pathways that are implicated in cell migration and metastasis [46]. To examine how these compounds affect migration, we performed wound-healing assays with Capan1 and CD18/HPAF cells. Cells were plated and allowed to grow to 90% confluence and wounds were introduced with a pipette tip. These plates were then treated with either vehicle or compound #1 or #6 (50 μM) and wound healing was monitored for 48 h. As shown in Figure 6A, both compound #1 (middle panel) and #6 (lower panel) reduced the migration of Capan1 cells compared to vehicle control. Analyses of the wound closure show that the compounds reduced wound closure in Capan1 and CD18/HPAF cells at 24 h and had ˜60% reduction compared to control at 48 h (Figure 6B).

We also examined the dose-dependent effects of both compounds on cell migration in both Capan1 and CD18/HPAF cells. Briefly, the above experiment was conducted with either vehicle or increasing concentrations of compound #1 or #6 and their effects on wound closure was determined at 48 h. Results in Figure 6C showed that treatment with compound #1 in a dose-dependent manner reduced migration of both Capan1 (left panel) and CD18/HPAF (right panel) cells. Furthermore, Capan1 cells appeared to be more sensitive to compound #1 than CD18/HPAF cells. More than 80% reduction in wound closure was observed when Capan1 cells were treated with 75 μM compound #1 compared to vehicle-treated cells, while ˜60% reduction in wound closure was detected in the CD18/HPAF cells with the same treatment. Although compound #6 was not as potent as compound #1, it also showed dose-dependent inhibition of migration of both Capan1 and CD18/HPAF cell lines (Figure 6D), with maximum inhibition of ˜60% and ˜40% inhibition respectively. These results indicate that compounds #1 and #6 both block cell migration in pancreatic cancer cells.

We validated the inhibitory effect of compound #1 and #6 on cell mobility using transwell assay. Capan1 and CD18/HPAF cells were pre-treated for 1 h with either vehicle control, or compound (#1 or #6 at 50 μM), re-seeded into transwells and evaluated for the compounds’ effects on the migration of cells through a barrier with 8-μM pores. As shown Supplementary Figure 2, the migration of both Capan1 and CD18/HPAF cells were significantly inhibited by the compound #1 and #6 (*p* < 0.001, *n* = 4).

### Inhibition of cell viability following treatment with compounds #1 and #6

**Figure 7:**
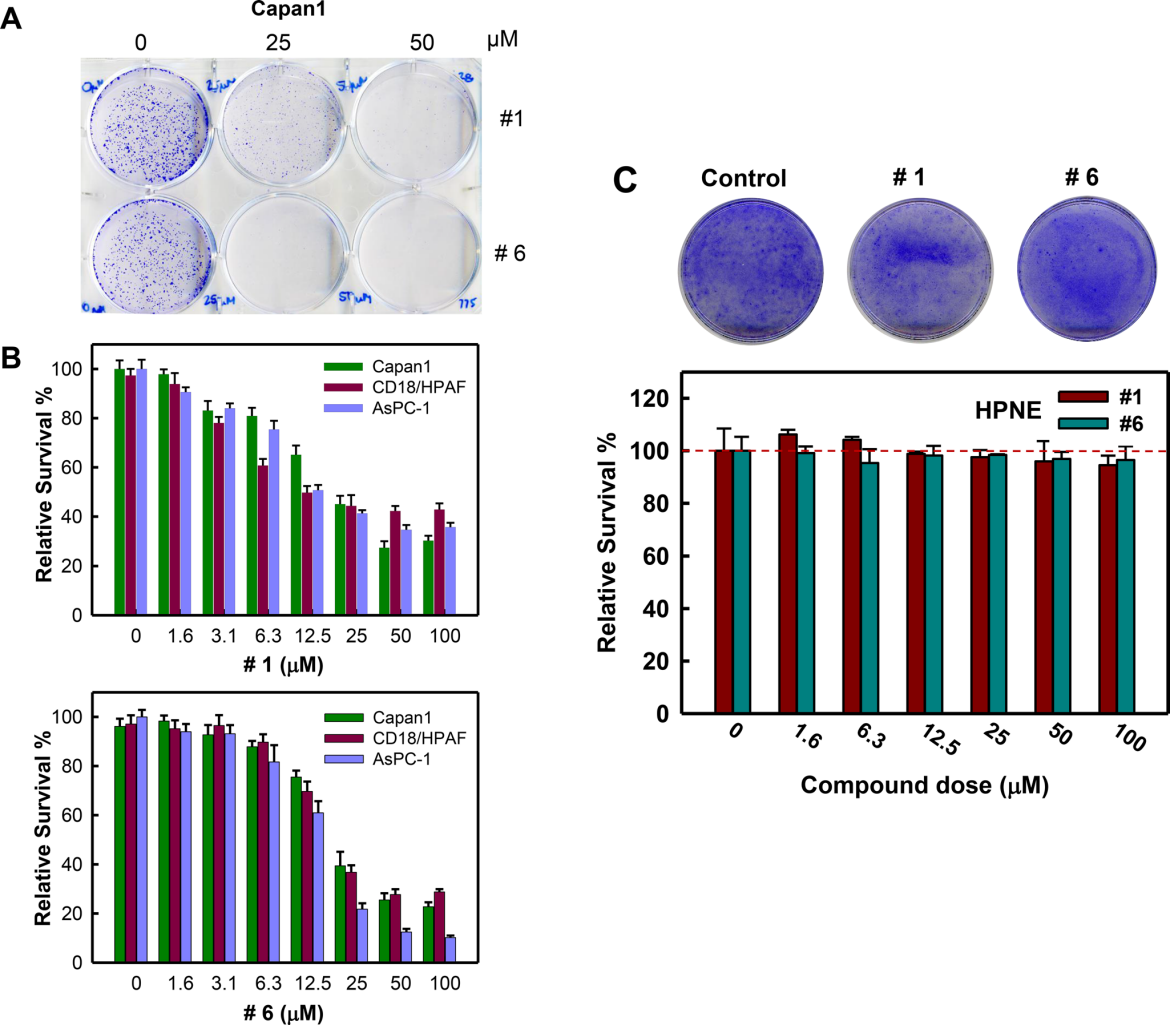
Compounds #1 and #6 inhibit clonogenic survival of pancreatic cancer cells Pancreatic cancer cells (2 × 10^3^) in 6-well plates were treated with vehicle, 25 μM or 50 μM of either compound #1 or #6 for 7 days and examined for colonies. (**A**) Representative sample dishes from the clonogenic assay are shown. (**B**) Viability of the indicated pancreatic cancer cells treated with increasing amounts of either compound #1 (upper panel) or #6 (lower panel) were quantified using PrestoBlue reagent, as described in Materials and Methods, and the obtained results were normalized to vehicle control. Results are shown as mean ± S.D. from two sets of experiments in duplicate samples. (**C**) HPNE cells were treated with increasing concentrations of compound #1 (green bars) and #6 (blue bars) for 7 days and assessed for survival as described above. Upper panel: representative images of HPNE cells treated with vehicle control, 50 μM compound #1 or 50 μM compound #6 for 7 days. Lower panel: amounts of the HPNE cells treated with compound #1 or #6 were assessed using ImageJ and obtained survival data was normalized to vehicle control. The results are shown as mean ± S.D. from two sets of experiments in duplicate samples.

Rac1 activity has been shown to be important to survival of cancer cells [42, 43]. We assessed the effect of the compounds on cell survival using a clonogenic assay. As shown in Figure 7A, treatment with compound #1 or #6 for 7 days resulted in decreased survival of Capan1 cells compared to vehicle control. We next examined these compounds for their dose-dependent effects on the viability of pancreatic cancer cells. We used Capan1, CD18/HPAF, and AsPC-1 cell lines, which were previously shown to have elevated Rac1 level/activity compared to normal pancreatic cells [34]. Following 5-day treatment with increasing concentrations of either compound #1 or #6 (0–100 μM), the viability of each cell line was determined. Figure 7B (upper panel) showed that compound #1 reduced cell viability in a dose-dependent manner and ˜65% inhibition was observed in all cell lines with 50 μM of compound #1 treatment. Similarly, compound #6 (Figure 7B, lower panel) also showed dose-dependent effects on cell viability in all cell lines and ˜75% inhibition was observed at 50 μM. The EC_50_ values were determined through curve fitting and are summarized in Table 1.

**Table 1:** EC_50_ values for compounds #1 and #6 for pancreatic cancer cell viability

Cell Line	Compound #1 (μM)	Compound #6 (μM)
AsPC-1	19.1 ± 2.5	14.7 ± 0.9
Capan1	21.7 ± 2.4	24.2 ± 1.8
CD18/HPAF	22.5 ± 4.7	23.5 ± 2.2

Pancreatic cancer cells were seeded in 96-well plates at 3 × 10^3^ cells/well for AsPC-1 and 2 × 10^3^ cells/well for Capan1 and CD18/HPAF in culture medium. Cells were treated with increasing concentrations of compound #1 or #6 (0, 1.6, 3.1, 6.3, 12.5, 25, 50 and 100 μM) and incubated for 7 days at 37°C. Viabilities of the treated cells were determined using PrestoBlue reagent as described in *Material and Methods*, and the obtained results were normalized to vehicle control. Half-maximal effective concentration (EC_50_) for the inhibition of clonogenic survival of the indicated cells by each compound was determined using SigmaPlot 11.0 software. The results were shown as mean ± S.D. from two sets of experiments in duplicate samples (*n* = 4).

Next, we examined the effect of compounds #1 and #6 on the survival of normal pancreatic ductal cells (HPNE) in a clonogenic assay. HPNE cells were treated for 7 days with increasing concentrations of either compound #1 or #6 (0 – 100 μM) and cell survival was assessed. As shown in Figure 7C, both compounds had little to no effect on the survival of HPNE cells even at the highest concentrations compared to controls. Collectively, these results suggest that compounds #1 and #6 inhibit the survival of pancreatic cancer cells with little effects on the survival of normal HPNE cells.

## DISCUSSION

It is well established that the hyper-activation of Rac1 signaling pathways is associated with numerous cancer-associated processes, including proliferation, motility, and survival, in multiple cancer cell types [42, 47–51]. In pancreatic cancers, Rac1 hyper-activation has also been implicated in the development and maintenance of Ras-mediated tumorigenesis [19, 52]. We have previously observed both elevated Rac1 level and elevated Rac1 activity in pancreatic cancer cells compared to normal pancreatic ductal cells [34], thus making hyper-activated Rac1 a promising therapeutic target for pancreatic cancers.

Unlike Ras proteins, activating mutations in Rac1 rarely drive the activation of the Rac1 pathway [12, 13, 16, 17], with a recently characterized activating mutation being Rac1 P29S, identified in melanoma [53, 54]. Overexpression of Rac1 and/or its GEFs has been shown to drive hyper-activation of Rac1 and its downstream pathways [21–25]. This has led to the development of Rac1 inhibitors that block GEF binding to Rac1. However, these have shown varied efficacy in different cell lines and types. This may be due to different tumors utilizing multiple GEFs to activate Rac1. In pancreatic cancers, more than 70% of tumors overexpressed the Rac1 GEFs Tiam1 and Vav1 [16, 17]. These GEFs have different binding modes to Rac1 and current Rac1-GEF inhibitors have shown limited efficacy against both GEFs [30, 55] indicating the need for additional chemotypes for targeting Rac1.

Our objective in this study was to identify inhibitors of Rac1 with previously unexplored core structures. Towards this goal, a virtual high-throughput screening campaign was undertaken with the 100,000-member ChemBridge chemical library. The docking sphere was selected to include the nucleotide-binding site of Rac1. Post-docking analyses and follow up studies identified two Rac1 inhibitors, compounds #1 and #6, which preferentially clustered to the nucleotide-binding site of Rac1. Cell-free and cell-based studies showed that both compounds reduced PAK1-binding to active Rac1 in a dose-dependent manner with nanomolar potency.

A nucleotide-binding fluorescence polarization assays showed compound #1 inhibits binding of fluorescently labeled GDP to Rac1 in a dose-dependent manner. This suggests that the biochemical mechanism for loss of PAK1 binding to Rac1 by compound #1 could be through the nucleotide-binding site. However, impaired nucleotide binding could also be caused by displacement of Mg^2+^, a key cofactor in Rac1 nucleotide binding or by competing for GEF binding or through unknown allosteric mechanisms [36, 37]. Additional biochemical assays as well as structural determination of Rac1 in complex with each compound (#1 and #6) are needed to clarify the exact mechanisms of inhibition.

We also evaluated the ability of these compounds to block the complex formation of PAK1 with intracellular Rac1. Our results showed that both compounds were able to dose-dependently inhibit the endogenous Rac1 activity in EGF-stimulated CD18/HPAF cells. Both compounds had EC_50_ in the low micromolar range for inhibition of PAK1 binding to Rac1 present in the EGF-stimulated CD18/HPAF cells, suggesting that compounds #1 and #6 will be effective in the inhibition of sustained hyper-activation of Rac1 seen in pancreatic cancer cells.

Selectivity studies in Figure 4 show that the compounds #1 and #6 inhibit formation of the Rac1-PAK1 complex but not the formation of the other two GTPases complexes, Cdc42-WASP and RhoA-Rhotekin. Consistently, while compound #1 dose-dependently inhibited the binding of mant-GDP to Rac1, it had little effect on the binding of mant-GDP to Cdc42, even though there is considerable structural and functional similarity with Rac1. Additional biochemical and cell-based studies are needed to further characterize the source of the observed selectivity of these compounds for Rac1.

Rac1 has been implicated as a cell cycle regulator and is known to promote cancer cell proliferation and survival [42, 43]. Our studies show that both compounds #1 and #6 were able to inhibit the viability of pancreatic cancer cells at micromolar concentrations. Furthermore, compound #1 showed a better growth inhibition profile when compared to EHT1864 (See Supplementary Figure 1B).

Selectively for cancer cells over normal cells is a critical issue for anticancer agents. We thus examined the effect of these compounds on the survival of both pancreatic cancer cells and immortalized cells derived from normal pancreas (HPNE). Our studies indicate that both compound #1 and #6 had a significant impact on the survival of pancreatic cancer cell lines but no such effect was observed with HPNE cells (See Figure 7). The mechanistic basis for the observed difference could be attributed to the addiction of cancer cells to Rac1 signaling. Inhibition of Rac1 activity therefore has a greater negative effect on pancreatic cancer cell survival over HPNE normal pancreatic cells. These observations indicate that both compounds could be utilized as viable non-toxic therapeutic agents for cancer treatment.

Rho GTPases, in particular Rac1, have long been recognized as key regulators of the actin cytoskeleton and cell migration [47]. Since cell motility plays a significant role in invasion and metastasis of cancer cells, we investigated the effects of compounds #1 and #6 on cell migration. Our results show that both compound #1 and #6 markedly inhibit migration of pancreatic cancer cells compared to controls, while compound #1 is more potent in the effect (See Figure 6). Additional studies examining the ability of compounds #1 and #6 to suppress specific Rac1-dependent cytoskeleton rearrangements, such as lamellipodia formation, are needed to clarify mechanism specific inhibition and to further explore these compounds as anti-metastatic therapeutics.

In summary, the present study describes the discovery of two small molecule Rac1 inhibitors with previously unexplored core structures. We provide evidence that both compounds block Rac1-PAK1 complex formation possibly by blocking nucleotide association to Rac1. Additionally, we show that both compounds preferentially down-regulate Rac1 activity compared to Cdc42 and RhoA in pancreatic cancer cells. The compounds also affect Rac1-regulated processes such as cell proliferation and cell migration. We also show that neither compound significantly impaired survival of normal pancreatic cells even at high concentrations. We conclude that compounds #1 and #6 are validated Rac1 inhibitors that are suitable for hit-to-lead optimization.

## MATERIALS AND METHODS

### Computational screen

A digital copy of a 100,000 member ChemBridge chemical library (San Diego, CA) was screened for compounds that could fit into the GTP-binding pocket of Rac1 (PDB Code: 3TH5). Compounds were docked using Molegro Virtual Docker on the Holland Computing Center's Sandhills Cluster using a docking sphere with a 9Å radius. The cavity representing the nucleotide binding site was identified using a ray-tracing algorithm. The docking sphere was centered over the nucleotide binding site and the screen was executed using GPU accelerated algorithm. The docking grid was set to a resolution of 0.2 Å. Each compound was allowed three socking runs with 256 simultaneous evaluations using 2.0 Å Tabu clustering to ensure high conformational diversity between poses. All poses were constrained to the 3D space of the binding cavity. Poses were then re-evaluated using a more comprehensive scoring function, the Molegro re-rank score. The compounds were then ranked based on their re-ranked score. Structures that ranked in the top 1% (1,000 structures) were considered for post-docking analysis. Poses with re-rank scores outside the 99% confidence interval were recommended for bench analysis. For post-docking analysis, ACD Percepta software was used to assess ADMET and physicochemical properties. Identified compounds were obtained from ChemBridge for further testing.

### Fluorescence polarization assay to assess mant-GDP binding to Rac1 and determination of IC_50_ and K_i_ values

All measurements were made on a 384-well, low-volume, black, round-bottom polystyrene NBS microplate (Corning, New York, NY) using a SpectraMax M5 plate reader (Molecular Devices, Sunnyvale, CA). Polarization values were measured at excitation wavelength 360 nm and emission wavelength 440 nm. Polarization was then normalized to percent bound fraction. Binding affinities were determined using 100 nM N-methylanthraniloyl (mant)-GDP (Life Technologies, Carlsbad, CA) and increasing concentrations of His-Rac1 (0.015 nM–150 μM) in assay buffer [20 mM Tris-HCl (pH 8.0), 50 mM NaCl, 10 mM EDTA, and 1 mM MgCl_2_]. Readings were taken after a 10-min incubation.

IC_50_ values were determined using increasing concentrations of 2 μl of each compound and incubating with 17 μL His-Rac1 (250 nM) for 1 h. Then 1 μL of mant-GDP (100 nM) was added to the reaction mixture and measurements were taken after a 10-min incubation. The data were then fitted using a nonlinear least squares fit to a single site-binding model (SigmaPlot 11.0) to determine IC_50_ values. The K_i_ values were determined using the Coleska-Wang equation [56]. Identical techniques were used to determine the apparent binding affinities and K_i_ values for Cdc42 and RhoA. Incubation times were increased to 30 min. mant-GDP concentration was 50 nM and protein concentration was 200 nM for all competition assays.

### Cell culture and treatment

Human pancreatic cancer cell lines AsPC-1, CD18/HPAF and Capan-1 were obtained from the American Type Culture Collection (Manassas, VA) and maintained in Dulbecco's Modified Eagle's medium containing 10% fetal bovine serum. HPNE cells are primary human pancreatic ductal cells immortalized using hTERT, the catalytic subunit of human telomerase [57]. HPNE cells were maintained in Medium D medium, which contains 3 parts of high glucose DMEM (Life Technologies, Carlsbad, CA), 1 part of M3F (INCELL, San Antonio, TX), 5% fetal bovine serum and 100 ng/ml recombinant EGF (Life Technologies) [57].

Rac1 specific inhibitor NSC23766 [21] was obtained from Tocris Biosciences (Ellisville, MO). Experimental compounds were purchased from ChemBridge (San Diego, CA). All compounds were dissolved in DMSO.

### Antibodies and recombinant proteins

All antibodies were obtained from Santa Cruz Biotechnology (Santa Cruz, CA) unless otherwise indicated. These included rabbit IgG for GAPDH (FL-335) and PAK1 (2602) (Cell Signaling); mouse IgG for Rac1 (23A8) (EMD Millipore), Cdc42 (B-8) and RhoA (26C4).

Recombinant PAK1-PBD (70-117aa), WASP-GBD (228-298aa), and Rhotekin-RBD (7-89aa) proteins for Rac1, Cdc42 and RhoA activity assays, respectively, were obtained from Addgene (Cambridge, NH) as glutathione S-transferase (GST) fusion proteins. All GST fusion proteins were purified as described previously [58]. Briefly, each protein was expressed in DH5α *E. coli* using 0.1 mM IPTG at 30°C overnight in a shaker incubator. Cell pellets were suspended in 10 mM Tris, pH 7.4, 1 mM phenylmethylsulfonyl fluoride (PMSF), and 1 mM DTT. Lysis was achieved by adding lysozyme (2 mg/mL), 13 mM NaCl, and 1% Triton-X 100 and incubated 50 min at 4°C followed by sonication. Samples were then clarified by centrifugation at 25,000 × g for 30 min. The supernatant was then incubated with glutathione resin (GE Healthcare) for 2 h at 4°C. The resin was washed and re-suspended in 20 mM HEPES, pH 7.6, 100 mM KCL, 1 mM EDTA, 1 mM DTT, and 10% glycerol.

Recombinant Rac1 (1-192aa) was obtained from DNASU (Tempe, AZ) as a His-tagged fusion protein. The protein was expressed in BL21 (Invitrogen) *E. coli* using 1 mM IPTG at 25°C overnight in a shaker incubator. The cell pellet was suspended in 50 mM sodium phosphate, pH 8.0, 300 mM NaCl, 10 mM MgCl_2_, 1 mM GDP (Chem Impex Intl.), 1 mM PMSF, and 1 mM DTT. Lysis was achieved by adding lysozyme (2 mg/mL) and 1% Triton-X 100 and incubated 40 min at 4°C followed by sonication. Samples were then clarified by centrifugation at 25,000 × g for 30 min. The supernatant was then incubated with nickel agarose resin (Sigma) for 2 h at 4°C. The resin was washed in buffer containing 10 mM imidazole and transferred to a gravity-flow poly-prep chromatography column (Bio-Rad). Rac1 was then eluted using 200 mM imidazole followed by dialysis in 20 mM Tris, pH 8.0, 50 mM NaCl, 1 mM MgCl_2_, 1 mM DTT, and 10% glycerol. Protein concentration was determined using a Bradford protein assay (Thermo Scientific) and purity was confirmed by SDS-PAGE.

Recombinant GST-Cdc42 (#12969) and recombinant 6XHis-RhoA (#73231) were received from Addgene. Proteins were expressed in DH5-Alpha cells using 1 mM IPTG at 18°C overnight in a shaker incubator in LB broth. Cell pellet was suspended in 30 mM Tris-HCl (pH 7.5), 100 mM NaCl, 5 mM MgCl_2_, and 2 mM 2-mercaptoethanol. GST-Cdc42 protein was lysed using emulsiflex, run over GSTrap 1 mL column (HiTrap) using AKTA Pure HPLC system, and eluted using buffer containing 20 mM reduced GSH. Fractions were then purified using anion exchange chromatography (30 mM Tris-HCl pH 7.5, 10 mM NaCl, 5 mM MgCl_2_, 2 mM 2-mercaptoethanol, eluted with buffer containing 1 M NaCl) followed by size exclusion chromatography (30 mM Tris-HCl pH 7.5, 100 mM NaCl, 5 mM MgCl_2_, 2 mM 2-mercaptoethanol). His-RhoA was lysed using above lysis buffer and run over HisTrap column (HiTrap) on AKTA Pure HPLC and eluted using buffer containing 1 M imidazole. Fractions were then purified using anion exchange and size exclusion as described above. Protein purity was confirmed by SDS-PAGE.

### Immunoblotting, immunoprecipitation and GST pull-down assays

Immunoblotting, immunoprecipitation and GST pull-down assays were performed as described previously [58–60]. Specific protein signals on Western blots were visualized by chemiluminescence exposed to x-ray film, scanned using EPSON Perfection 4490 PHOTO scanner and analyzed using the ImageJ analytical program (NIH, Bethesda, MD).

### Rac1, Cdc42 and RhoA activity assays

Rac1, Cdc42 and RhoA activity was assessed using a Rac1/Cdc42 assay kit (Upstate Biotechnology, Lake Placid, NY), as described previously [61, 62]. Briefly, CD18/HPAF cells were lysed at 4°C in 50 mM Tris-HCl (pH 7.5) containing 10 mM MgCl_2_, 1% TritonX-100, 1 mM EDTA, 1 mM EGTA, 2 mM DTT, 5 mM sodium pyrophosphate, 10 mM sodium 2-glycerolphosphate, 1 μg/ml aprotinin, 1 μg/ml leupeptin, 1 μg/ml pepstatin, 1 mM phenylmethylsulfonyl fluoride, 50 mM sodium fluoride, and 1 mM sodium vanadate. Cell lysates were incubated with agarose beads coated with GST-PAK1, GST-WASP, or GST-Rhotekin fusion protein for 1 h to capture GTP-bound Rac1, Cdc42, or RhoA, respectively. The obtained GTP-bound Rac1 (Rac1-GTP), Cdc42 (Cdc42-GTP), or RhoA (RhoA-GTP) was resolved on a 15% gel using SDS-PAGE and assessed by immunoblotting using an anti-Rac1, anti-Cdc42, or anti-RhoA antibody, as described by the manufacturer's instruction. As a negative control, CD18/HPAF cell lysates were incubated with 1 mM GDP at 30^°^C for 15 min and analyzed for Rac1 activity as instructed by the manufacturer.

Cell-free Rac1 activity assays were performed using 5 μg of purified His-Rac1 in 20 μl GTP binding buffer [20 mM Tris-HCl (pH 7.6), 100 mM NaCl, 2 mM MgCl_2_ and 1 mM DTT) with 1% BSA. For GTP-γS linearity tests, increasing concentrations of GTP-γS were added to GTP binding buffer containing His-Rac1, incubated for 10 minutes at room temperature and followed by 1 h incubation with GST-PAK1. GTP-bound Rac1 in the test tubes was assessed as described above. To examine the compounds’ effects on Rac1 activity *in vitro*, increasing concentrations of each compound was incubated with His-Rac1 for 1 h followed by the addition of GTP-γS (200 nM) for 10 min. GTP-bound Rac1 was then pulled-down using agarose beads coated with GST-PAK1, separated on a 15% gel using SDS-PAGE and assessed by immunoblotting. Apparent binding affinities, EC_50_, and IC_50_ values were determined by curve fitting the data as previously described.

### Growth kinetics

Growth kinetics was determined using AlamarBlue assay (Life Technologies, Carlsbad, CA) as described previously [44]. Cells (1.5 × 10^3^) in 96-well plates were incubated with 25 μM compound (#1 or #6) or vehicle control for 24, 72, or 120 h and determined for amount of cells using PrestoBlue reagent (Life Technologies, Carlsbad, CA). Fluorescent measurements were taken using a Spectramax M5 plate reader (MDS). Each experiment was repeated twice in triplicate.

### Cell viability assay

Cells were seeded in 96-well plates at 2 × 10^3^ cells/well for Capan1 and CD18/HPAF and at 3 × 10^3^ cells/well for AsPC-1 and HPNE in culture medium. Cells were treated with an increasing concentration of compound (1.6–100 μM) and incubated at 37°C for up to 7 days. Cell viability was determined using PrestoBlue reagent (Life Technologies, Carlsbad, CA) according to the manufacturer's protocol. Fluorescent measurements were taken using a Spectramax M5 plate reader (MDS). Each experiment was repeated twice in triplicate.

### Cell mobility examination

Wound healing migration assay: Capan1 and CD18/HPAF pancreatic cancer cells were seeded in a 6-well plate at 90% confluence and wounds were made down the central axis of each well using a pipette tip. Cells were treated with either compound or vehicle control (DMSO) in a dose-dependent manner. Cell migration was visualized at magnification 5× using an Axiovert40C scope (Zeiss) and photographed with a CoolPIX4300 camera (Nikon). Images were taken at 0, 24, and 48 h. Migration was analyzed using ImageJ analytical program (NIH, Bethesda, MD). Average wound closure was determined by averaging the measurements of 15 separate wound widths for each data set.

Transwell migration assay: The potential of cells to migrate was also assessed in transwell insert with 8.0 μm pore polycarbonate membrane (BD Biosciences, Franklin Lakes). Briefly, cells were pre-treated with either compound (50 μM) or vehicle control (DMSO) for 1 h, trypsinized and seeded onto the upper chambers of the trans-well (0.5 × 10^5^ cells/well) in serum-free DMEM medium with/without compound. The lower chambers of the transwell were filled with medium containing 10% FBS with/without compound. After incubation at 37°C and 5% CO_2_ for 24 h, cells on the upper surface of the filter were removed using a cotton swab, whereas cells invasive through the filter to the lower surface were fixed with 4% paraformaldehyde for 10 min and stained with 0.1% crystal violet for 30 min. After staining, the cells on the membrane were scanned using EPSON Perfection 4490 PHOTO scanner and analyzed using the ImageJ analytical program (NIH, Bethesda, MD).

### Clonogenic survival assays

Clonogenic survival assays were performed as described previously [63]. Briefly, pancreatic cancer cells were seeded at 2000 cells per well in 6-well plates in duplicate. Log-phase growing cells were incubated with DMSO as a vehicle control or compound #1 or #6 for 7–14 days until colonies formed. For HPNE normal pancreatic epithelial cells, cells were seeded in a 6-well plate at 30% confluence per well in duplicate. Log-phase growing HPNE cells were then incubated with DMSO as vehicle control or increasing concentrations of compound #1 or #6 for 7 days. The colonies were visualized by crystal violet staining and analyzed using ImageJ as described previously [64].

## SUPPLEMENTARY MATERIALS FIGURES AND TABLES



## References

[1] Etienne-Manneville S, Hall A (2002). Rho GTPases in cell biology. Nature.

[2] Heasman SJ, Ridley AJ (2008). Mammalian Rho GTPases: new insights into their functions from *in vivo* studies. Nat Rev Mol Cell Biol.

[3] Jaffe AB, Hall A (2005). Rho GTPases: biochemistry and biology. Annu Rev Cell Dev Biol.

[4] Zheng Y (2001). Dbl family guanine nucleotide exchange factors. Trends Biochem Sci.

[5] Karlsson R, Pedersen ED, Wang Z, Brakebusch C (2009). Rho GTPase function in tumorigenesis. Biochim Biophys Acta.

[6] Sahai E, Marshall CJ (2002). RHO-GTPases and cancer. Nat Rev Cancer.

[7] Vega FM, Ridley AJ (2008). Rho GTPases in cancer cell biology. FEBS Lett.

[8] Westwick JK, Lambert QT, Clark GJ, Symons M, Van Aelst L, Pestell RG, Der CJ (1997). Rac regulation of transformation, gene expression, and actin organization by multiple, PAK-independent pathways. Mol Cell Biol.

[9] Pai SY, Kim C, Williams DA (2010). Rac GTPases in human diseases. Dis Markers.

[10] Mack NA, Whalley HJ, Castillo-Lluva S, Malliri A (2011). The diverse roles of Rac signaling in tumorigenesis. Cell Cycle.

[11] Wertheimer E, Gutierrez-Uzquiza A, Rosemblit C, Lopez-Haber C, Sosa MS, Kazanietz MG (2012). Rac signaling in breast cancer: a tale of GEFs and GAPs. Cell Signal.

[12] Schnelzer A, Prechtel D, Knaus U, Dehne K, Gerhard M, Graeff H, Harbeck N, Schmitt M, Lengyel E (2000). Rac1 in human breast cancer: overexpression, mutation analysis, and characterization of a new isoform Rac1b. Oncogene.

[13] Engers R, Ziegler S, Mueller M, Walter A, Willers R, Gabbert HE (2007). Prognostic relevance of increased Rac GTPase expression in prostate carcinomas. Endocr Relat Cancer.

[14] Espina C, Cespedes MV, Garcia-Cabezas MA, Gomez del Pulgar MT, Boluda A, Oroz LG, Benitah SA, Cejas P, Nistal M, Mangues R, Lacal JC (2008). A critical role for Rac1 in tumor progression of human colorectal adenocarcinoma cells. Am J Pathol.

[15] Crnogorac-Jurcevic T, Efthimiou E, Capelli P, Blaveri E, Baron A, Terris B, Jones M, Tyson K, Bassi C, Scarpa A, Lemoine NR (2001). Gene expression profiles of pancreatic cancer and stromal desmoplasia. Oncogene.

[16] Guo X, Wang M, Jiang J, Xie C, Peng F, Li X, Tian R, Qin R (2013). Balanced Tiam1-rac1 and RhoA drives proliferation and invasion of pancreatic cancer cells. Mol Cancer Res.

[17] Fernandez-Zapico ME, Gonzalez-Paz NC, Weiss E, Savoy DN, Molina JR, Fonseca R, Smyrk TC, Chari ST, Urrutia R, Billadeau DD (2005). Ectopic expression of VAV1 reveals an unexpected role in pancreatic cancer tumorigenesis. Cancer Cell.

[18] Denicola G, Tuveson DA (2005). VAV1: a new target in pancreatic cancer?. Cancer Biol Ther.

[19] Heid I, Lubeseder-Martellato C, Sipos B, Mazur PK, Lesina M, Schmid RM, Siveke JT (2011). Early requirement of Rac1 in a mouse model of pancreatic cancer. Gastroenterology.

[20] Hirshberg M, Stockley RW, Dodson G, Webb MR (1997). The crystal structure of human rac1, a member of the rho-family complexed with a GTP analogue. Nat Struct Biol.

[21] Gao Y, Dickerson JB, Guo F, Zheng J, Zheng Y (2004). Rational design and characterization of a Rac GTPase-specific small molecule inhibitor. Proc Natl Acad Sci USA.

[22] Ferri N, Corsini A, Bottino P, Clerici F, Contini A (2009). Virtual screening approach for the identification of new Rac1 inhibitors. J Med Chem.

[23] Zins K, Lucas T, Reichl P, Abraham D, Aharinejad S (2013). A Rac1/Cdc42 GTPase-specific small molecule inhibitor suppresses growth of primary human prostate cancer xenografts and prolongs survival in mice. PLoS One.

[24] Montalvo-Ortiz BL, Castillo-Pichardo L, Hernandez E, Humphries-Bickley T, De la Mota-Peynado A, Cubano LA, Vlaar CP, Dharmawardhane S (2012). Characterization of EHop-016, novel small molecule inhibitor of Rac GTPase. J Biol Chem.

[25] Bouquier N, Vignal E, Charrasse S, Weill M, Schmidt S, Leonetti JP, Blangy A, Fort P (2009). A cell active chemical GEF inhibitor selectively targets the Trio/RhoG/Rac1 signaling pathway. Chem Biol.

[26] Bouquier N, Fromont S, Zeeh JC, Auziol C, Larrousse P, Robert B, Zeghouf M, Cherfils J, Debant A, Schmidt S (2009). Aptamer-derived peptides as potent inhibitors of the oncogenic RhoGEF Tgat. Chem Biol.

[27] Qiu RG, Chen J, Kirn D, McCormick F, Symons M (1995). An essential role for Rac in Ras transformation. Nature.

[28] Gao Y, Xing J, Streuli M, Leto TL, Zheng Y (2001). Trp of rac1 specifies interaction with a subset of guanine nucleotide exchange factors. J Biol Chem.

[29] Karnoub AE, Worthylake DK, Rossman KL, Pruitt WM, Campbell SL, Sondek J, Der CJ (2001). Molecular basis for Rac1 recognition by guanine nucleotide exchange factors. Nat Struct Biol.

[30] Chrencik JE, Brooun A, Zhang H, Mathews II, Hura GL, Foster SA, Perry JJ, Streiff M, Ramage P, Widmer H, Bokoch GM, Tainer JA, Weckbecker G (2008). Structural basis of guanine nucleotide exchange mediated by the T-cell essential Vav1. J Mol Biol.

[31] Surviladze Z, Waller A, Wu Y, Romero E, Edwards BS, Wandinger-Ness A, Sklar LA (2010). Identification of a small GTPase inhibitor using a high-throughput flow cytometry bead-based multiplex assay. J Biomol Screen.

[32] Shutes A, Onesto C, Picard V, Leblond B, Schweighoffer F, Der CJ (2007). Specificity and mechanism of action of EHT 1864, a novel small molecule inhibitor of Rac family small GTPases. J Biol Chem.

[33] Yan Y, Greer PM, Cao PT, Kolb RH, Cowan KH (2012). RAC1 GTPase plays an important role in gamma-irradiation induced G2/M checkpoint activation. Breast Cancer Res.

[34] Yan Y, Hein AL, Etekpo A, Burchett KM, Lin C, Enke CA, Batra SK, Cowan KH, Ouellette MM (2014). Inhibition of RAC1 GTPase sensitizes pancreatic cancer cells to gamma-irradiation. Oncotarget.

[35] Bishop AL, Hall A (2000). Rho GTPases and their effector proteins. Biochem J.

[36] Zhang B, Zhang Y, Wang Z, Zheng Y (2000). The role of Mg2+ cofactor in the guanine nucleotide exchange and GTP hydrolysis reactions of Rho family GTP-binding proteins. J Biol Chem.

[37] Menard L, Snyderman R (1993). Role of phosphate-magnesium-binding regions in the high GTPase activity of rac1 protein. Biochemistry.

[38] Haeusler LC, Blumenstein L, Stege P, Dvorsky R, Ahmadian MR (2003). Comparative functional analysis of the Rac GTPases. FEBS Lett.

[39] Fanger GR, Johnson NL, Johnson GL (1997). MEK kinases are regulated by EGF and selectively interact with Rac/Cdc42. EMBO J.

[40] Itoh RE, Kiyokawa E, Aoki K, Nishioka T, Akiyama T, Matsuda M (2008). Phosphorylation and activation of the Rac1 and Cdc42 GEF Asef in A431 cells stimulated by EGF. J Cell Sci.

[41] Hakoshima T, Shimizu T, Maesaki R (2003). Structural basis of the Rho GTPase signaling. J Biochem.

[42] Moore KA, Sethi R, Doanes AM, Johnson TM, Pracyk JB, Kirby M, Irani K, Goldschmidt-Clermont PJ, Finkel T (1997). Rac1 is required for cell proliferation and G2/M progression. Biochem J.

[43] Yoshida T, Zhang Y, Rivera Rosado LA, Chen J, Khan T, Moon SY, Zhang B (2010). Blockade of Rac1 activity induces G1 cell cycle arrest or apoptosis in breast cancer cells through downregulation of cyclin D1, survivin, and X-linked inhibitor of apoptosis protein. Mol Cancer Ther.

[44] Pessetto ZY, Yan Y, Bessho T, Natarajan A (2012). Inhibition of BRCT(BRCA1)-phosphoprotein interaction enhances the cytotoxic effect of olaparib in breast cancer cells: a proof of concept study for synthetic lethal therapeutic option. Breast Cancer Research and Treatment.

[45] Friedl P, Wolf K (2003). Tumour-cell invasion and migration: diversity and escape mechanisms. Nat Rev Cancer.

[46] Chan AY, Coniglio SJ, Chuang YY, Michaelson D, Knaus UG, Philips MR, Symons M (2005). Roles of the Rac1 and Rac3 GTPases in human tumor cell invasion. Oncogene.

[47] Parri M, Chiarugi P (2010). Rac and Rho GTPases in cancer cell motility control. Cell Commun Signal.

[48] Callery MP, Chang KJ, Fishman EK, Talamonti MS, William Traverso L, Linehan DC (2009). Pretreatment assessment of resectable and borderline resectable pancreatic cancer: expert consensus statement. Ann Surg Oncol.

[49] Aref A, Berri R (2012). Role of radiation therapy in the management of locally advanced pancreatic cancer. J Clin Oncol.

[50] de Lange SM, van Groeningen CJ, Meijer OW, Cuesta MA, Langendijk JA, van Riel JM, Pinedo HM, Peters GJ, Meijer S, Slotman BJ, Giaccone G (2002). Gemcitabine-radiotherapy in patients with locally advanced pancreatic cancer. Eur J Cancer.

[51] Epelbaum R, Rosenblatt E, Nasrallah S, Faraggi D, Gaitini D, Mizrahi S, Kuten A (2002). Phase II study of gemcitabine combined with radiation therapy in patients with localized, unresectable pancreatic cancer. J Surg Oncol.

[52] Wertheimer E, Kazanietz MG (2011). Rac1 takes center stage in pancreatic cancer and ulcerative colitis: quantity matters. Gastroenterology.

[53] Hodis E, Watson IR, Kryukov GV, Arold ST, Imielinski M, Theurillat JP, Nickerson E, Auclair D, Li L, Place C, Dicara D, Ramos AH, Lawrence MS (2012). A landscape of driver mutations in melanoma. Cell.

[54] Krauthammer M, Kong Y, Ha BH, Evans P, Bacchiocchi A, McCusker JP, Cheng E, Davis MJ, Goh G, Choi M, Ariyan S, Narayan D, Dutton-Regester K (2012). Exome sequencing identifies recurrent somatic RAC1 mutations in melanoma. Nat Genet.

[55] Worthylake DK, Rossman KL, Sondek J (2000). Crystal structure of Rac1 in complex with the guanine nucleotide exchange region of Tiam1. Nature.

[56] Nikolovska-Coleska Z, Wang R, Fang X, Pan H, Tomita Y, Li P, Roller PP, Krajewski K, Saito NG, Stuckey JA, Wang S (2004). Development and optimization of a binding assay for the XIAP BIR3 domain using fluorescence polarization. Anal Biochem.

[57] Lee KM, Nguyen C, Ulrich AB, Pour PM, Ouellette MM (2003). Immortalization with telomerase of the Nestin-positive cells of the human pancreas. Biochem Biophys Res Commun.

[58] Yan Y, Black CP, Cowan KH (2007). Irradiation-induced G2/M checkpoint response requires ERK1/2 activation. Oncogene.

[59] Sarkaria JN, Busby EC, Tibbetts RS, Roos P, Taya Y, Karnitz LM, Abraham RT (1999). Inhibition of ATM and ATR kinase activities by the radiosensitizing agent, caffeine. Cancer Res.

[60] Hall-Jackson CA, Cross DA, Morrice N, Smythe C (1999). ATR is a caffeine-sensitive, DNA-activated protein kinase with a substrate specificity distinct from DNA-PK. Oncogene.

[61] Wei Q, Adelstein RS (2002). Pitx2a expression alters actin-myosin cytoskeleton and migration of HeLa cells through Rho GTPase signaling. Mol Biol Cell.

[62] Cook JA, Albacker L, August A, Henderson AJ (2003). CD28-dependent HIV-1 transcription is associated with Vav, Rac, and NF-kappa B activation. J Biol Chem.

[63] Kuo PL, Hsu YL, Cho CY (2006). Plumbagin induces G2-M arrest and autophagy by inhibiting the AKT/mammalian target of rapamycin pathway in breast cancer cells. Mol Cancer Ther.

[64] Cai Z, Chattopadhyay N, Liu WJ, Chan C, Pignol JP, Reilly RM (2011). Optimized digital counting colonies of clonogenic assays using ImageJ software and customized macros: comparison with manual counting. Int J Radiat Biol.

[65] Seshacharyulu P, Ponnusamy MP, Rachagani S, Lakshmanan I, Haridas D, Yan Y, Ganti AK, Batra SK (2015). Targeting EGF-receptor(s) - STAT1 axis attenuates tumor growth and metastasis through downregulation of MUC4 mucin in human pancreatic cancer. Oncotarget.

