# Supplementary material for: Discovery and characterization of small molecule Rac1 inhibitors
Source: Oncotarget. 2017 Mar 29;8(21):34586–600. doi: 10.18632/oncotarget.16656 (PMC5470993; doi:10.18632/oncotarget.16656)
Supplement: Supplementary file 1 [file oncotarget-08-34586-s001.pdf]

Discovery and characterization of small molecule Rac1 inhibitors

Supplementary Materials

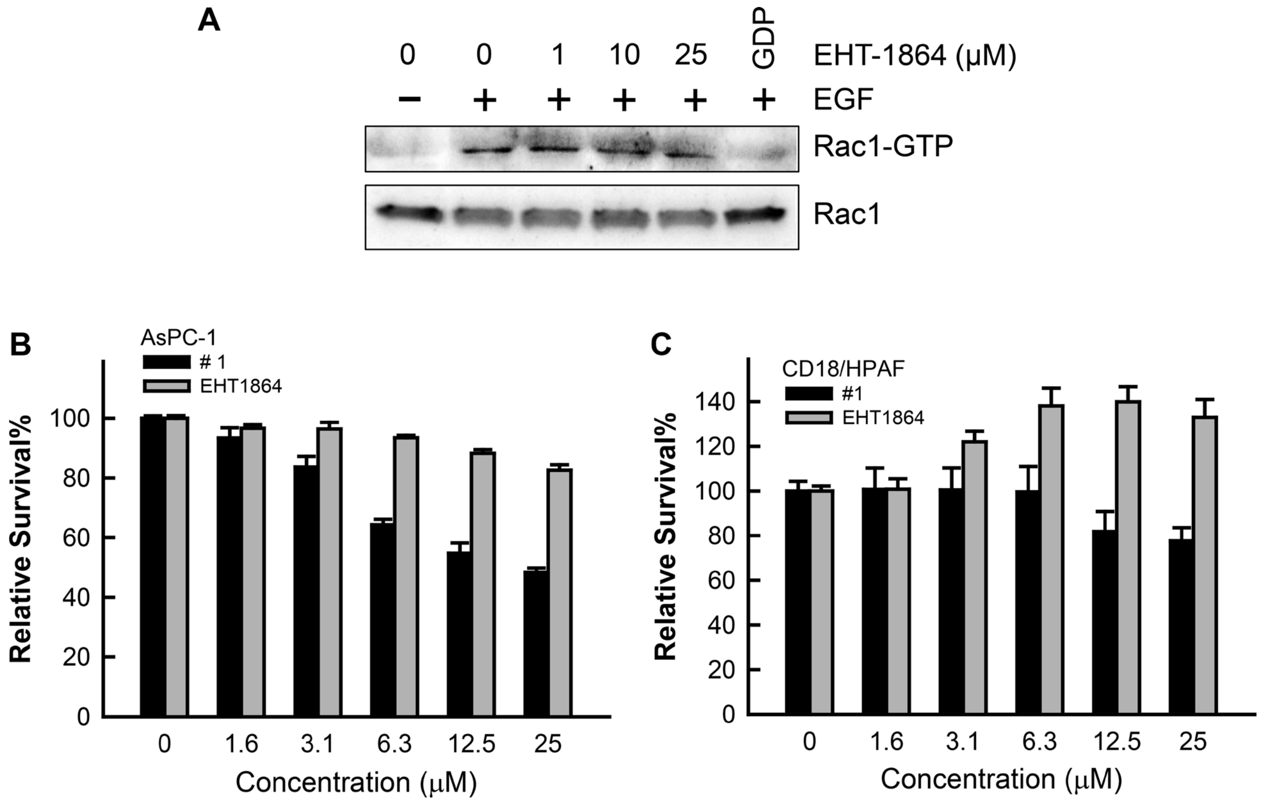

Supplementary Figure 1: Comparison of compound #1 and EHT1864 for their effects on survival of pancreatic cancer cells. (**A**) CD18/HPAF cells were serum starved in the medium containing 0.3% FBS for 24 h, incubated for 2 h with increasing concentrations of EHT1864 and then stimulated with EGF (100 ng/ml) for 5 min. Rac1 activity (Rac1-GTP level) in the samples were determined by Rac1 assay. Total protein level of Rac1 is included as a control. (**B**) AsPC1 cells (4×10^3) and (**C**) CD18/HPAF cells were seeded in 96-well plates and incubated with increasing concentrations of either compound #1 or EHT1864 for 3 days. Cellular viability was determined by AlamarBlue assay and normalized to vehicle control. Results are shown as mean ± S.D. of two sets of experiments done in triplicate samples.

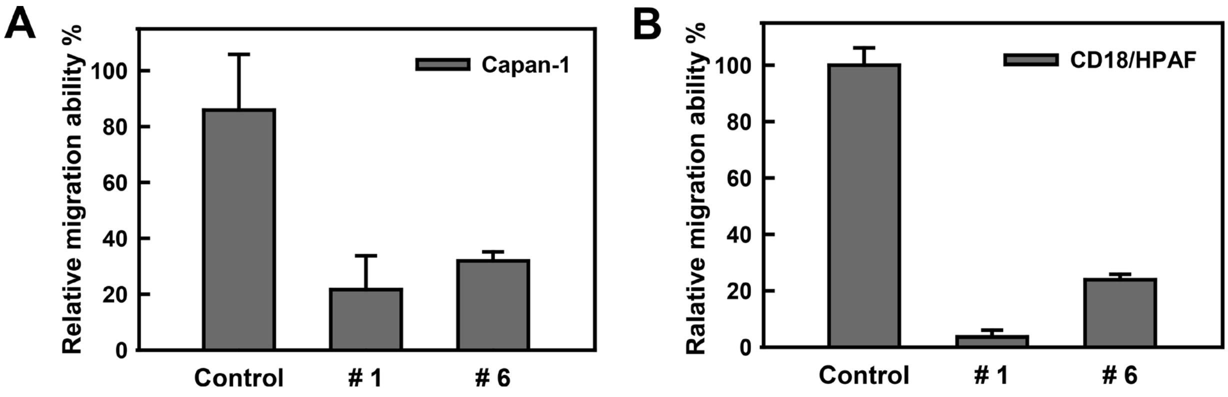

Supplementary Figure 2: Compounds #1 and #6 inhibit the migration potential of pancreatic cancer cells. Capan1 (**A**) and CD18/HPAF (**B**) cells were pretreated with DMSO vehicle control (0.1%), compound #1 (50 µM) or compound #6 (50 µM) for 1 h, trypsinized and seeded (0.5×10^5 cells/well) onto the upper chambers of the trans-well insert with 8.0 µm pore polycarbonate membrane, which were filled with serum-free DMEM medium with/without compound (50 µM). The lower chambers of the transwell were filled with medium containing 10% FBS with/without compound (50 µM). After incubated at 37°C and 5% CO_2 for 24 h, the cells migrated through the filter to the lower surface were fixed with 4% paraformaldehyde for 10 min and stained with 0.1% crystal violet for 30 min, visualized by scanning using EPSON Perfection 4490 PHOTO scanner and quantified using the ImageJ analytical program. Migration data was normalized to relative migration in vehicle control and shown as mean ± S.D. from two sets of experiments in duplicate samples. $*p < 0.001$ ($n = 4$), significant difference between the cells incubated with the compound #1 or #6 and the cells in vehicle control.
